# A Color- and Geometric-Feature-Based Approach for Denoising Three-Dimensional Cultural Relic Point Clouds

**DOI:** 10.3390/e26040319

**Published:** 2024-04-05

**Authors:** Hongjuan Gao, Hui Wang, Shijie Zhao

**Affiliations:** 1 School of Information Engineering, Ningxia University, Yinchuan 750021, China; 12022131976@nxu.edu.cn (H.W.); 12022132009@nxu.edu.cn (S.Z.); 2 Ningxia Key Laboratory of Artificial Intelligence and Information Security for Channeling Computing Resources from the East to the West, Yinchuan 750021, China; 3 Collaborative Innovation Center for Ningxia Big Data and Artificial Intelligence Co-Founded by Ningxia Municipality and Ministry of Education, Yinchuan 750021, China

**Keywords:** 3D point cloud, cultural relics, denoising, graph signal processing, Markov graph model, prior probability distribution, maximum a posteriori (MAP) estimation

## Abstract

In the acquisition process of 3D cultural relics, it is common to encounter noise. To facilitate the generation of high-quality 3D models, we propose an approach based on graph signal processing that combines color and geometric features to denoise the point cloud. We divide the 3D point cloud into patches based on self-similarity theory and create an appropriate underlying graph with a Markov property. The features of the vertices in the graph are represented using 3D coordinates, normal vectors, and color. We formulate the point cloud denoising problem as a maximum a posteriori (MAP) estimation problem and use a graph Laplacian regularization (GLR) prior to identifying the most probable noise-free point cloud. In the denoising process, we moderately simplify the 3D point to reduce the running time of the denoising algorithm. The experimental results demonstrate that our proposed approach outperforms five competing methods in both subjective and objective assessments. It requires fewer iterations and exhibits strong robustness, effectively removing noise from the surface of cultural relic point clouds while preserving fine-scale 3D features such as texture and ornamentation. This results in more realistic 3D representations of cultural relics.

## 1. Introduction

Three-dimensional laser scanning technology has become increasingly popular in various fields of society, such as digitization, virtual display, and virtual restoration of cultural relics. However, the acquisition process of cultural relic point clouds often results in noise in geometry and color due to the inherent limitations of 3D laser scanners or depth cameras. This noise can be caused by occlusion resulting from various view angles, reflective materials, dust on the surface of objects, light intensities, and the operation of scanning personnel [1]. The cultural relic point cloud surface typically contains significant fine details, such as ornamentation or textures, which can be intertwined with surface noise. Effectively removing noise from the surface of the cultural relic point cloud while preserving the fine-scale 3D features is a significant challenge.

In order to acquire a high-precision 3D model of a cultural relic with realistic texture, it is essential to remove noise from the raw 3D point cloud. The noise in the point cloud can be divided into two categories based on their distribution: surface noise and outliers [2]. Each outlier will usually be far away from the surface of the point cloud with a sparse neighborhood, which means that they are easy to remove using methods such as the boxplot method or special software. However, eliminating surface noise presents a greater challenge as it is often closely intertwined with the underlying surface of the 3D point cloud. This is especially true when the surface of the 3D point cloud features texture and ornamentation.

To obtain clean point clouds for further processing, various surface smoothing techniques have been developed in the past two decades. These techniques include filtering-based methods [3,4,5,6], moving least squares (MLS)-based methods [7,8], locally optimal projection (LOP)-based methods [9,10], non-local methods [11,12,13], and sparsity-based methods [14,15,16,17]. Although these methods have been successful in achieving excellent denoising effects for 3D models with smooth surfaces, they have not yielded satisfactory results for point clouds of cultural relics. This often results in over-smoothing and the loss of surface details. Striking a balance between preserving fine details and achieving effective denoising with these methods is challenging.

In recent years, several methods have been proposed for denoising point clouds, including the graph feature learning method [18,19,20,21,22,23,24,25,26] and the deep learning method [27,28,29,30,31,32,33]. 

The effectiveness of deep learning in denoising point clouds depends heavily on factors such as the geometric structure, the scale of the data, and the noise characteristics of the training set. When faced with an unknown scene or limited data, the method based on deep learning may not necessarily outperform traditional methods. For instance, a model trained using commonly available 3D point clouds may experience a significant decrease in performance when applied to point clouds of cultural relics, which are considered to be rare samples.

Graph-based denoising methods utilize graph filters to remove noise from point clouds represented by graphs [34]. Previous methods such as graph Laplacian regularization (GLR) [20] and the feature graph learning [23] algorithm have shown promising results in inferring the underlying graph structure of clean point clouds. However, these methods primarily rely on geometric priors, making it challenging to achieve effective denoising while preserving fine detail.

We raise an interesting question: if color perception information is added to guide the graph signal processing, can a balance between denoising effectiveness and detail preservation be achieved?

To investigate this, we propose a novel 3D point cloud denoising method based on graph signal processing specifically designed for cultural relic point clouds. Our contributions are twofold. First, we incorporate not only geometric information such as 3D coordinates and surface normals but also color distribution as a feature. The use of a multi-modal representation for vertex features leads to superior denoising performance. Second, we introduce a 3D point cloud simplification module to dynamically adjust the number of 3D point clouds to reduce the running time of the denoising algorithm.

This paper is organized as follows: In Section 2, we introduce previous point cloud denoising methods. In Section 3, we describe the basic concepts of graph signal processing. In Section 4, we provide the details of our proposed method, which mainly focuses on surface noise removal. In Section 5, we present the experimental results and discussion. Finally, we present our conclusions.

## 2. Related Work

Point cloud denoising techniques can be divided into two main types: outlier removal techniques and surface noise smoothing techniques. Outlier removal is a relatively straightforward process, as outliers are usually distinct from other data points and can be easily identified and removed. On the other hand, surface noise removal can be more challenging, as surface noise is often random and irregular and requires more sophisticated techniques to be removed. In this paper, we will primarily focus on surface noise removal methods.

Filtering-based methods: Filtering-based methods were initially used for 2D image smoothing and were later extended to denoise 3D point clouds [2]. These methods assume that the noise on the surface of point clouds is high-frequency noise, and they use filters that target vertices or face normals. Early approaches utilized Laplacian smoothing or improved Laplacian transform based on vertex positions to denoise triangular meshes. However, this often resulted in the excessive smoothing of surface features and was not effective when dealing with large amounts of noise. In recent years, filtering-based methods have been significantly improved. Notable examples include guided normal filtering [3,4] and rolling guidance normal filtering [5], which have demonstrated successful denoising effects in practical applications [6]. Nevertheless, a major drawback of these methods is that the normal filtering process tends to blur the small-scale features of the 3D model surface, resulting in the over-smoothing of 3D models with intricate surface details.

MLS-based and LOP-based methods: Early in the development of denoising technology, moving least squares (MLS) and local optimal projection (LOP) methods were well-known and popular denoising methods. However, their denoising effect is limited, and they are no longer the mainstream methods. MLS-based methods [7] approximate the point cloud using a smooth surface and project the points from the input point cloud onto the fitted surface. These methods are unstable in cases of a low sampling rate or high curvature and are highly sensitive to outliers [8]. LOP-based methods [9] aim to find the best possible solution to represent the underlying surface within a local region of the search space while ensuring an even distribution across the input point cloud. However, these methods can suffer from over-smoothing [10].

Non-local methods: Non-local methods [11,12,13] establish self-similarity among surface patches in the point cloud by solving an optimization problem. However, these methods often suffer from high computational complexity when searching for non-local similar patches.

Sparsity-based methods: Sparsity-based methods [14,15,16] transform the denoising problem of a 3D point cloud into an optimization problem. This is achieved by obtaining a sparse representation of the surface normal by minimizing the number of non-zero coefficients with sparsity regularization. To preserve the sharp features of the 3D point cloud, either the $L_{0}$ or $L_{1}$ norm is used. It should be noted that sparsity-based methods tend to give better denoising results when the noise is small. However, for high noise levels, these methods can suffer from either over-sharpening or over-smoothing [17].

Graph-based methods: Graph-based denoising methods [18,19,20,21,22,23,24,25,26] transform the problem of removing noise into a graph-constrained optimization problem and perform noise removal through the structure and connectivity of the graph. However, a drawback of these approaches is that they often misestimate the local surface by relying solely on the geometry information of the vertices. In addition, the performance of graph-based denoising methods remains unstable for highly noisy point clouds.

Deep learning methods: Deep learning denoising methods [27,28,29,30,31,32,33,34,35] train an end-to-end neural network to remove noise. During the training stage, the model learns the mapping between noisy points and clean clouds. In the testing stage, the trained model is used to denoise point clouds with similar noise characteristics and geometric shapes. Deep learning methods are more effective at denoising and preserving fine features. However, these methods require a large volume of training data, making them time-consuming and impractical for unknown scenes. In addition, optimizing and improving the efficiency of the algorithm is also an important consideration.

Several alternative denoising methods have been proposed by other scholars. For instance, there is a point cloud denoising algorithm based on a method library [36], as well as a laser point cloud denoising method that uses principal component analysis (PCA) and surface fitting [37]. However, these methods often encounter the common issue of inadequate denoising of sharp edges, resulting in excessive smoothing.

Recently, some scholars have proposed deep-unfolding denoising [38,39,40] and quantum-based denoising [41,42], which have achieved competitive results compared to state-of-the-art image denoising tasks. How to draw on the ideas of these methods to denoise the point cloud is a very valuable research work in the future. 

## 3. Preliminaries

### 3.1. Graph Signal and Graph Laplacian

In this section, we present a brief overview of fundamental concepts in graph signal processing. We define an undirected weighted graph for a vertex set of cardinality |V|=N, where the edge set E connects vertices of the form $\left(v_{i},v_{j}\right)\in V$. Each edge is assigned a non-negative weight $w_{i,j}$, and the adjacency matrix W is a N×N real matrix with values ranging from 0 to 1. The combinatorial graph Laplacian is defined as L:=D−W, where D represents the degree matrix of the graph G, with $d_{ij}=\overset{N}{\underset{j=1}{\sum }}w_{ij}$ denoting the degree of each vertex.

### 3.2. Graph Laplacian Prior

Graph signal data reside on the vertices of a graph, which include 3D coordinates, normal vectors, and color information on a 3D point cloud. A graph signal z is considered to be smooth with respect to the topology of G if it satisfies the following conditions:

(1) $$z^{T}Lz=\sum_{i=1}^{N}\sum_{j=1}^{N}w_{i,j}{\left(z_{i}-z_{j}\right)}^{2}<є,$$

where є is a positive scalar, and the Laplacian matrix L is a symmetric positive semi-definite matrix. The larger $w_{ij}$ is, the more similar $z_{i}$ and $z_{j}$ are and the smaller the value of $z^{Τ}Lz$ is. 

Formula (1) forces signal z to adapt to the topology of G, which is referred to as graph Laplacian regularization (GLR), also known as graph signal smoothness prior. By minimizing the graph Laplacian regularization term, the signal can be smoothed. This prior is used in our paper to remove the surface noise, as discussed in Section 4.

If we consider L(z) as a function for signals z, then reweighted prior is redefined as

(2) $$z^{T}L\left(z\right)z=\sum_{i=1}^{N}\sum_{j=1}^{N}w_{i,j}\left(z_{i},z_{j}\right)\cdot {\left(z_{i}-z_{j}\right)}^{2},$$

where $w_{i,j}\left(z_{i},z_{j}\right)=\exp\left\{-{\left(z_{i}-z_{j}\right)}^{2}/\sigma^{2}\right\}$, and $w_{i,j}\left(z_{i},z_{j}\right)$ is the (*i, j*)-th element of the corresponding adjacency matrix W.

## 4. The Proposed Method

Considering a point cloud contaminated by noise  Y ′ with a Gaussian noise distribution, our basic strategy can be viewed from the theory of graph signal processing; our goal is to move noisy points to the underlying surface to generate a clean point cloud Y.

**Figure 1:**
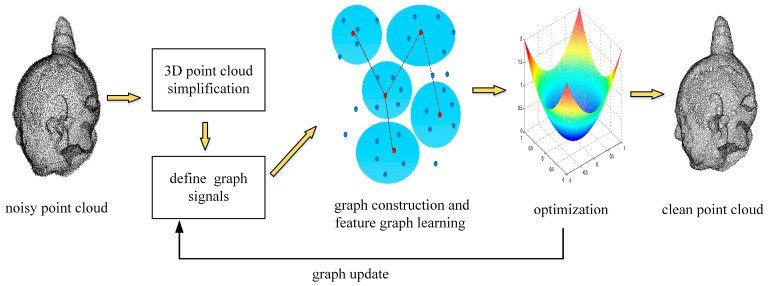
The proposed denoising approach.

Figure 1 illustrates the implementation of our denoising approach, which consists of the following four modules:

- Simplification of 3D point cloud: reducing the collection of points to a smaller subset that retains its fundamental topology, thereby reducing the running time of the denoising algorithm;
- Definition of graph signals: interpreting the geometric and color information of the vertices in the input point clouds as graph signals; the geometric information includes geometry coordinates and normal vectors;
- Graph construction and feature graph learning: defining local patches within the point cloud and constructing a graph model with Markovian properties; using a feature graph learning scheme to determine edge weights and solving a maximum a posteriori (MAP) estimation problem with GLR as the signal prior;
- Application of an optimization algorithm to enforce smoothness on the graph signal: we alternately optimize the feature metric matrix M by minimizing the GLR, and M and noisy point cloud are updated alternately until the algorithm converges, and finally, we obtain the clean point cloud.

### 4.1. Three-Dimensional Point Cloud Simplification

High-precision 3D artifact point clouds usually have many points, inevitably leading to high computational complexity and long processing times during denoising. Therefore, it is necessary to simplify the raw data to a more appropriate size without affecting the denoising effect. We use the method described in [43] for simplifying point clouds. The simplification process is divided into the following four steps:

- A bounding box for the point cloud is created. A local kd−tree consisting of 27 cubes of size 3 × 3 × 3 is constructed. The advantage of using this form to organize the points in the point cloud is that the neighborhood points and leaf nodes of the given point can be accurately identified.
- Five feature indexes are calculated to extract features from the point cloud. These five feature indexes include the curvature feature index, the density feature index, the edge feature index, the terrain feature index, and the 3D feature index, denoted as a, b, c, d, and e, respectively. The advantage of this multiple-feature indexing approach is that it can deal with different types of point clouds and discover more intrinsic characteristics of the point cloud.
- The weights of the five feature indexes are calculated using the analytic hierarchy process (AHP) method based on data features. Assuming that $w_{i}$ is the weight index of feature indexes a, b, c, d, and e, the quantification result of point p is $z_{p}=\left[a_{p},b_{p},c_{p},d_{p},e_{p}\right]{\left[\omega_{a},\omega_{b},\omega_{c},\omega_{4},\omega_{5}\right]}^{T}$.
- Points with larger z-values are identified as feature points, and points with smaller z-values are identified as non-feature points. All feature points form a simplified point cloud. According to the kd−tree constructed earlier, if there is no feature point in each leaf node, the non-feature point closest to the center of gravity of the node is selected to be added to the simplified point cloud.

### 4.2. Defining the Graph Signal by Combining Geometry and Color

**Figure 2:**
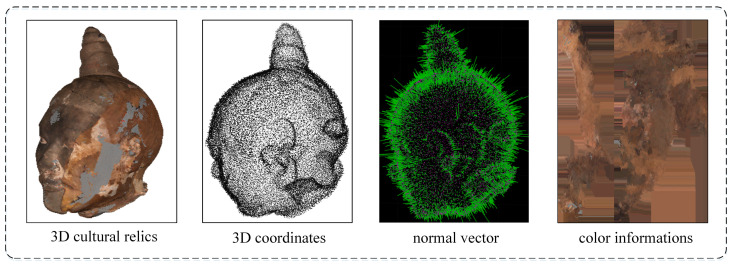
Geometry and color information of a vertex in a point cloud.

Color, an important piece of information of a point cloud, has been used for 3D model retrieval [44] and point cloud segmentation [45,46]. The combination of color and geometry can positively affect graph construction, which is more semantically meaningful than using geometry alone [47]. In this paper, we aim to use both the color and geometry attributes of a vertex in the point cloud to investigate their crucial role in denoising. Figure 2 illustrates the color and geometry information of the point cloud.

We constructed a k-NN graph with Markovian properties, where each vertex is connected to its *k*-nearest neighbors by connecting edges with associated weights. In addition to using the 3D coordinates and normal vector of the vertices as signals, we added the color attributes as graph signals. The feature vector of a vertex in the graph is denoted as

(3) $$s_{i}=[P_{i}\;N_{i}\;C_{i}]\in R^{9},$$

where 3D coordinates $P_{i}=[x_{i},y_{i},z_{i}]\in R^{3}$, normal vector $N_{i}=\left[n_{x}^{i},n_{y}^{i},n_{z}^{i}\right]\in R^{3}$, and RGB color information $C_{i}=\left[c_{r}^{i},c_{g}^{i},c_{b}^{i}\right]\in R^{3}$ for vertex $v_{i}$. 

**Figure 3:**
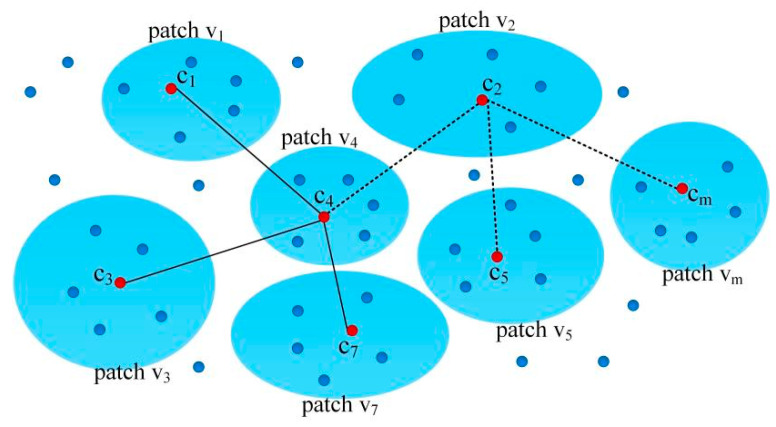
Illustration of the patch $v_{2}$, $v_{7}$ and their adjacent patches.

The normal vector is one of the important properties of the points in a point cloud. The normal vector of a point cloud is the orientation of each point in a point cloud. For example, the direction of the normal vector in Figure 3 points to the outside of the surface of the point cloud. The normal vector of a point cloud is usually a 3D vector that describes the normal properties of the point cloud surface, such as the flatness, curvature, and normal variation in the point cloud surface.

### 4.3. Constructing a Graph Based on Self-Similarity Theory

We followed the method described in [26] to define the local patches in a point cloud. These patches may overlap with each other. We assumed that these patches were self-similar [48] and established connections between corresponding points, forming a k-NN graph. We considered each local patch of the point cloud as a matrix, which had a low rank. Consequently, the problem of denoising the point cloud can be reformulated as a task for minimizing the rank of the matrix.

In this study, we used a uniform sampling method to select m center points $c_{i}\in R^{3}$ from point cloud *Y*. For each center point $c_{i}$, we used the k-nearest-neighbor (k-NN) [49] algorithm to identify k-nearest-neighbor points in terms of Euclidean distance. A patch $v_{i}$ is a set of points that is composed of one center point $c_{i}$ and k nearest neighbors. The number of nearest neighbors m was determined based on an empirical value, denoted as m≤N,(k+1)m≥N, where N is the number of points in the point cloud Y. As a result, we obtained m local patches from the point cloud Y, as shown in Figure 3. The collection of all points within these patches is referred to as a patch set, denoted as $V\in R^{\left(k+1\right)m\times 3}$.

Then, we identified ε adjacent patches for patch $v_{i}$. We used the k-NN algorithm to find the ε nearest center point for the center point $c_{i}$ of patch $v_{i}$ in a set of patches. The ε patches that the ε nearest center points are located in are recognized as adjacent patches of $v_{i}$. As shown in Figure 3, ε=3, and the adjacent patches of $v_{2}$ are $v_{4}$, $v_{5}$, and $v_{m}$. For a point $p_{i}\in v_{s}$, there exists a nearest corresponding point $p_{j}\in v_{t}$, and the Euclidean distance between $p_{i}$ and $p_{j}$ is the smallest.

In the process of constructing the local patches mentioned above, each patch is only related to its adjacent patches. The vertices in the patch are only connected to the corresponding vertices in the adjacent patch. As a result, these vertices and edges form a graph model with Markov properties.

### 4.4. Graph Feature Learning

We aim to calculate an optimal Mahalanobis distance $\delta_{i,j}$ for the given signals, which are represented as length-9 vectors of relevant features in a graph. We assumed two sets, $\emptyset_{k}\left(i\right)$ and $\emptyset_{k}\left(j\right)$, which denote the *k*-nearest neighbors to vertices $v_{i}$ and $v_{j}$, respectively. If $p_{j}\in \emptyset_{k}\left(i\right)$ or $p_{i}\in \emptyset_{k}\left(j\right)$, then

(4) $$\delta_{i,j}=-\frac{{\left\|P_{i}-P_{j}\right\|}^{2}}{\theta_{P}^{2}}-\frac{{\left\|N_{i}-N_{j}\right\|}^{2}}{\theta_{N}^{2}}-\frac{{\left\|C_{i}-C_{j}\right\|}^{2}}{\theta_{C}^{2}},$$

where $N_{i}$ and $N_{j}$ represent the normal vector of vertices $v_{i}$ and $v_{j}$; $C_{i}$ and $C_{j}$ represent the color information of vertices $v_{i}$ and $v_{j}$; $\theta_{P}$ and $\theta_{N}$ represent the relative contribution of the 3D coordinates and normal vectors in the constructed graph; and $\theta_{C}$ represents the relative contribution of color.

Defining $s_{i}={\left[p_{i},N_{i},C_{i}\right]}^{T}$, we express (4) in matrix form as

(5) δi,j=(si−sj)T[1θP20001θN20001θC2](si−sj),

where $s_{i}-s_{j}$ is the feature difference between the two connected nodes $p_{i}$ and $p_{j}$. The appropriate parameters $\theta_{P}$, $\theta_{N}$, and $\theta_{C}$ play an important role in achieving good denoising performance. How to determine these parameters is the next aspect to consider. 

The 3D coordinates, normal vector, and color information are features of different scales. In this context, we used the Mahalanobis distance as a measure of the similarity between the two signals. The Mahalanobis distance $\delta_{ij}$ is written as

(6) $$\delta_{i,j}={\left(s_{i}-s_{j}\right)}^{T}M\left(s_{i}-s_{j}\right),$$

where $M\in R^{k\times k}$ is the Mahalanobis distance matrix, which is a measure of the relative importance of individual features in the calculation of $\delta_{ij}$.

In the context of a graph, the edge weight $w_{i,j}\left(s_{i},s_{j}\right)$ represents the similarity of the signals between two samples. We define the edge weight $w_{i,j}\left(s_{i},s_{j}\right)$ using the Gaussian kernel, a commonly used method, which guarantees that the resulting graph Laplacian matrix L is positive semi-definite.

(7) $$w_{i,j}(s_{i},s_{j})=\exp\{-\delta_{ij}\},$$

The GLR, expressed in Formula (2), is redefined as

(8) $$s^{T}L\left(M\right)s=\sum_{i=1}^{N}\sum_{j=1}^{N}\exp\left\{-{\left(s_{i}-s_{j}\right)}^{T}M\left(s_{i}-s_{j}\right)\right\}\cdot {\left(s_{i}-s_{j}\right)}^{2}.$$

### 4.5. Optimization Algorithm

We considered the solution of a clean point cloud as a feature graph learning problem. As discussed in [23], we minimized the GLR and determined the appropriate underlying graph based on signal *z*. 

Additionally, we assumed a point cloud with added noise, namely

(9) $$Y^{\prime }=Y+E,$$

where $Y^{\prime }\in R^{N\times 3}$ denotes the 3D coordinates of the point cloud with added noise, $Y\in R^{N\times 3}$ denotes the 3D coordinates of the clean point cloud, and $E\in R^{N\times 3}$ denotes the white Gaussian noise (AWGN) [50] that appears near the underlying surface. The AWGN has zero mean and standard deviation.

(10) $$E∼N\left(0,\sigma^{2}I\right).$$

Given a noisy set $V^{\prime }$, the goal is to minimize the noise and obtain a noiseless set V. This is achieved by applying the maximum a posteriori criterion, which involves finding the most probable V given the observed $V^{\prime }$.

(11) $${\tilde{V}}_{\mathrm{MAP}}(V^{\prime })=\arg\underset{Y}{\max}P(V^{\prime }|V)\;P(V),$$

where P(V) is the prior probability distribution of V, and $P(V^{\prime }|V)$ is the likelihood function.

In the case of additive Gaussian white noise, the likelihood function is defined as follows:

(12) $$P(V^{\prime }|V)=P(Y^{\prime }|Y)=\exp\left\{\left(-1/\left(2\sigma^{2}\right)\right){\left\|Y-Y^{\prime }\right\|}_{F}^{2}\right\},$$

where ${\left\|\cdot \right\|}_{F}^{2}$ is the Frobenius norm.

If G is a graph with Markov properties [51], and GLR is taken as the prior probability distribution of the set V, then the following is evident:

(13) $$P(V)=\exp\left\{-\beta \mathrm{tr}(V^{⊤}L\left(M\right)V)\right\},$$

where $\beta ={\left(2\pi \right)}^{-\frac{n-1}{2}}{\left({\left|L\left(M\right)\right|}^{*}\right)}^{\frac{1}{2}}$ and M is the Markov distance matrix.

The denoising formula can be obtained by combining (11)–(13).

(14) $$\begin{array}{l} \underset{Y,M}{\min}{\left\|Y-Y^{\prime }\right\|}_{F}^{2}+(2\sigma^{2}\beta )\mathrm{tr}\left(V^{⊤}L\left(M\right)V\right),\\ s.t.\;M≻0;\;\mathrm{tr}(M)\leq C^{*}. \end{array}$$

It should be noted that $C^{*}$ is a constraint parameter closely related to the algorithm performance. 

Denoising a 3D point cloud is an iterative process. In the first iteration, M is initialized with the identity matrix. Then, the Laplacian matrix L(M) is computed, and the conjugate gradient method [52] is used to solve it. In the subsequent iteration, M is updated, and the optimization problem of M is solved using the near-end gradient method (PG) [53]. The values of M and Y are updated alternately until they converge.

The optimization algorithm is presented in Algorithm 1.
**Algorithm 1: Optimization algorithm**    Input: Noisy point cloud $Y^{\prime }$, number of patches *m*, number of nearest neighbors *k*,    number of adjacent patches ε, trace constraint $C^{*}$.    Output: Denoised point cloud *Y*.1     Initialize *Y* with $Y^{\prime }$;2     for iter = 1, 2,… do3     estimate normal for *Y;*
4     initialize m empty patches *V;*5     find the adjacent ε patches;6     initialize *M* with identity matrix;7     compute the feature distance $s_{i}-s_{j}$ for each vertex pair(*i,j*);8     solve *M*;9     compute adjacency matrix *W* over all patches;10         compute Laplacian matrix *L*;11         solve *Y* with (14);12    end

## 5. Experiment Results and Analysis

### 5.1. Experiment Environment and Dataset

Our method was implemented on a desktop computer running MS Windows 10. The computer was equipped with an Intel^®^ Core™ i9-9900k CPU (3.60 GHz), 64 GB of RAM, and two GeForce RTX 2070 GPUs. We used MATLAB R2019b programming for the implementation.

**Figure 4:**
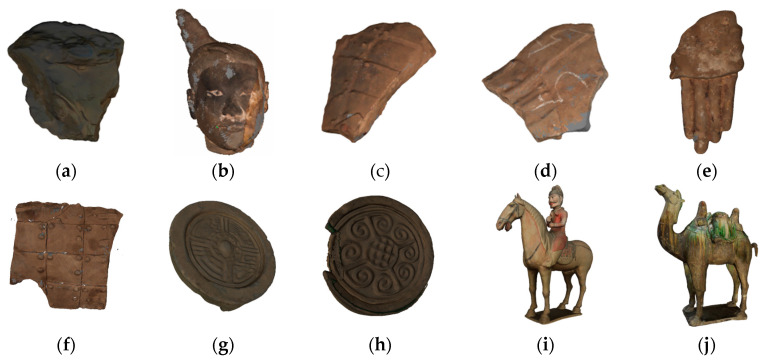
Three-dimensional point clouds of cultural relics: (**a**–**f**) terracotta warrior fragments numbered G3-I-b-70, 4#yt, G10-52, G10-46-5, G3-I-C-94, and G10-11-43(47); (**g**,**h**) Qin Dynasty tiles numbered Q002789 and Q003418; (**i**,**j**) Tang tri-color Hu terracotta sculptures numbered H73 and H80.

To demonstrate the state of the art of our approach, we performed experiments on 3D point clouds of terracotta warrior fragments, tiles from the Qin Dynasty, and Tang tri-color Hu terracotta sculptures, as shown in Figure 4. We achieved the best performance of the algorithm by selecting the optimal parameters. We repeated the experiments thirty times and calculated the average results for three metrics: *SNR*, *MSE*, and running time.

### 5.2. Evaluation Metrics

The evaluation of the denoising results was performed using visual effects, *SNR*, and *MSE*, following recent point cloud denoising research. Let us assume that the real point cloud and the predicted point cloud are denoted as $U={\left\{\;u^{i}\right\}}_{i=1}^{N_{1}}$ and $V={\left\{\;v^{i}\right\}}_{i=1}^{N_{2}}$, respectively.$\;u^{i},\;v^{i}\in R^{3}$, and $N_{1}$ and $N_{2}$ may not be equal here.

To measure the fidelity of the denoising result, we used MSE, which is the minimum absolute error sum of the normal direction difference between the noisy point cloud and the denoised point cloud. A lower *MSE* value indicates a better denoising effect. The calculation of the *MSE* is as follows:

(15) $$MSE=\frac{1}{2N_{1}}\sum_{u_{i}\in U}\underset{v_{j}\in V}{\min}{\left\|u_{i}-v_{j}\right\|}_{2}^{2}+\frac{1}{2N_{2}}\sum_{v_{i}\in V}\underset{u_{j}\in U}{\min}{\left\|v_{i}-u_{j}\right\|}_{2}^{2}$$

The SNR is a measure of the signal-to-noise ratio in a 3D point cloud, usually expressed in decibels. A higher signal-to-noise ratio indicates better denoising reliability of the algorithm. The SNR can be calculated using the following formula:

(16) $$SNR=10\log\frac{1/N_{2}\sum_{v_{i}\in V}{\left\|v_{i}\right\|}_{2}^{2}}{MSE}$$

### 5.3. Algorithm Performance Analysis

#### 5.3.1. The Effect of Parameters on Algorithm Performance

Our algorithm has four main parameters: the number of patches *m*, the number of points in each patch *k*, the number of nearest neighboring patches ε, and the constraint parameter $C^{*}$. Among these parameters, $C^{*}$ has a significant impact on the denoising effect. Therefore, it is important to determine the optimal value for *C*. To do so, we can first choose an initial value based on experience and then explore values around this initial value with a certain step size to determine the optimal value.

In this study, we analyzed the effect of parameter values on the MSE and SNR under Gaussian white noise with standard deviations *σ* = 0.02, *σ* = 0.05, and *σ* = 0.1. To illustrate this, we chose the 3D fragment numbered G3-I-b-70 as our experimental data source. 

**Figure 5:**
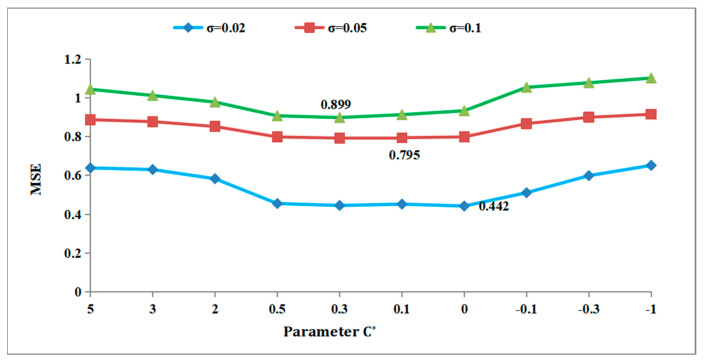
Effect of $C^{*}$ on MSE for G3−Ib−70.

As illustrated in Figure 5, the blue line represents the MSE value for noise with a *σ* of 0.02. The red trend line represents the MSE value for noise with a *σ* of 0.05, while the green trend line represents the MSE value for noise with a *σ* of 0.1. When the value of $C^{*}$ is 0, the minimum MSE value on the blue line is 0.442, indicating the algorithm’s optimal denoising effect of the algorithm at this point. Similarly, when the value of $C^{*}$ is 0.1, the minimum MSE on the red line is 0.795, signifying the best denoising effect. Lastly, with a $C^{*}$ value of 0.3, the minimum MSE on the green line is 0.899, denoting the optimal denoising effect of the algorithm at this particular point.

**Figure 6:**
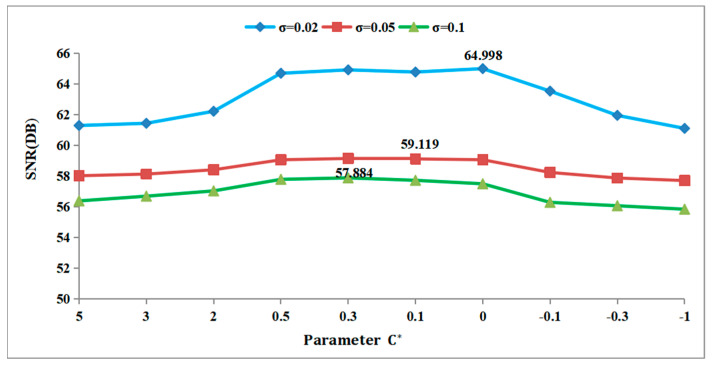
Effect of $C^{*}$ on SNR for G3−I−b−70.

As shown in Figure 6, as the noise levels vary, and the $C^{*}$ value changes, the denoising effect of the algorithm, as indicated by the SNR value, remains consistent with the MSE value. When the value of $C^{*}$ is 0.3, the maximum SNR on the green line is 57.884, indicating the best denoising effect. Similarly, when the value of $C^{*}$ is 0.1, the maximum SNR value on the red line is 59.119, indicating the optimal denoising effect of the algorithm at this point. Finally, with a $C^{*}$ value of 0, the maximum SNR on the blue line is 64.998, indicating the optimal denoising effect of the algorithm at this particular point.

#### 5.3.2. Ablation Experiment

In this study, we present the experimental results for two proposed methods: one using only geometry and the other using both geometry and color. We identified four main parameters that gave the best results for the proposed approach, as shown in Table 1.

The algorithm was evaluated based on four quantitative indicators: SNR, MSE, iterations, and running time. For the sake of clarity, the subjective results of the comparison between the proposed algorithm using only geometry and combined geometry and color are presented in Table 2. To illustrate this, we chose the 3D fragment numbered 4#yt as our experimental data source.

Table 2 shows that the SNR and MSE of the denoising algorithm with the combined geometry and color serving as graph signals outperform the geometry-only approach. Furthermore, the inclusion of color information does not result in a significant increase in iterations or running time.

As shown in Table 3, the 3D point cloud is simplified by reducing the number of points from 58,380 to 30,000. When *σ* = 0.02, the number of iterations of the denoising algorithm is reduced by 3, and the running time of the algorithm is reduced by 129 s. Similarly, when *σ* = 0.05, the number of iterations of the denoising algorithm is reduced by 9, and the running time is reduced by 731 s. These results show that when *σ* is less than 0.1, the value of SNR and MSE is almost unchanged. Therefore, in low-noise scenarios, it is advisable to first simplify the high-precision 3D cultural relic model obtained using scanning and then proceed with denoising. 

When *σ* = 0.1, the number of iterations of the denoising algorithm is reduced by 41, and the running time of the algorithm is reduced by 3718 s. Similarly, when *σ* = 0.2, the number of iterations of the denoising algorithm is reduced by 77, and the running time is reduced by 7899 s. It can be seen that the running time of the algorithm is greatly reduced, and the denoising effect is not significantly weakened when the point cloud is simplified to a reasonable size. Therefore, for some real-time application scenarios, it is necessary to simplify the point cloud before denoising.

#### 5.3.3. Iterations

**Figure 7:**
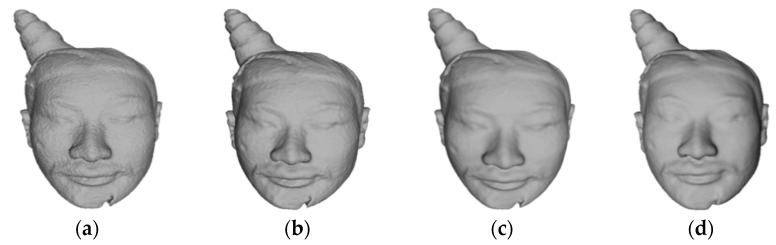
The denoising effect of our algorithm in different iterations on 4#yt: (**a**) description of the noisy input; (**b**) description of the third iteration; (**c**) description of the sixth iteration; (**d**) description of the tenth iteration.

This section presents the subjective results of the proposed combined color and geometry denoising approach. Figure 7a shows the presence of numerous noise points on the surface of 4#yt, resulting in an uneven surface. Subsequently, in Figure 7b, after the third iteration, the sharp noise points on the surface of the 3D model appear smoother. Furthermore, Figure 7c shows that as the number of iterations increases to 6, the rough areas on the surface of the 3D model gradually become smoother. Finally, Figure 7d shows that when the number of iterations reaches 10, the surface noise points are effectively eliminated.

The experimental results in Table 2 show that the number of iterations of the algorithm is influenced by the noise level. For *σ* = 0.02, the denoising algorithm needs 4 iterations; for *σ* = 0.05, the denoising algorithm needs 11 iterations. It can be observed that as the noise level increases, more iterations are required. Conversely, when the noise level is low, the proposed method shows the advantages of fewer iterations and a faster convergence speed.

#### 5.3.4. Robustness

The robustness of the proposed method was tested under different noise levels. Gaussian white noise was added to the clean 3D model in reverse, and the denoising effect of the proposed method was verified. In the experiment, the standard deviation *σ* of the white Gaussian noise was set to 0.02, 0.05, 0.1, and 0.2.

**Figure 8:**
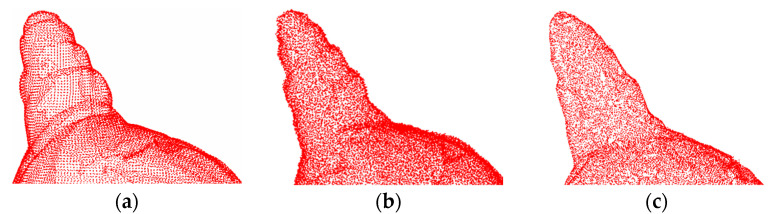
The denoising effect of 4#yt with Gaussian white noise with *σ* = 0.02 added: (**a**) description of the clean point cloud; (**b**) description of the noisy input; (**c**) description of the denoising effect.

**Figure 9:**
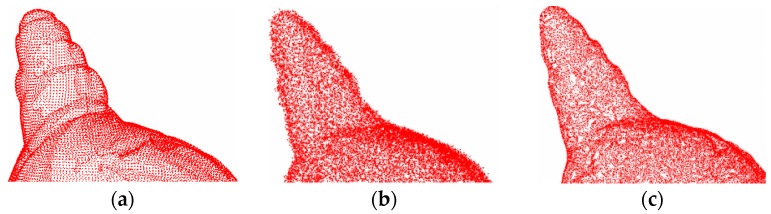
The denoising effect of 4#yt with Gaussian white noise with *σ* = 0.05 added: (**a**) description of the clean point cloud; (**b**) description of the noisy input; (**c**) description of the denoising effect.

Figure 8 and Figure 9 show the denoising effects of the proposed method after adding Gaussian white noise with *σ* = 0.02 and *σ* = 0.05, respectively. The experimental results indicate that at a low noise level, the surface smoothness of this method is almost indistinguishable from that of clean point clouds.

**Figure 10:**
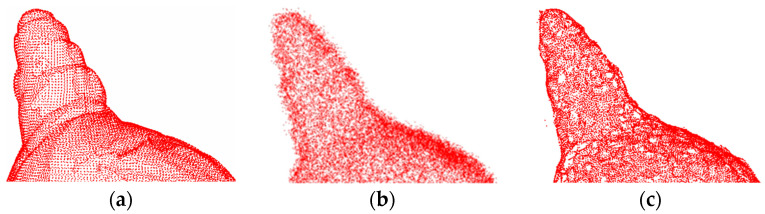
The denoising effect of 4#yt with Gaussian white noise with *σ* = 0.1 added: (**a**) description of the clean point cloud; (**b**) description of the noisy input; (**c**) description of the denoising effect.

**Figure 11:**
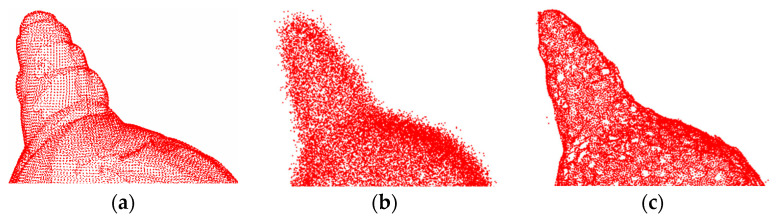
The denoising effect of 4#yt with Gaussian white noise with *σ* = 0.2 added: (**a**) description of clean point cloud; (**b**) description of the noisy input; (**c**) description of the denoising effect.

Figure 10 and Figure 11 show the denoising effects of the proposed method after adding Gaussian white noise with *σ* = 0.1 and *σ* = 0.2, respectively. Clearly, the proposed method exhibits excellent denoising performance even at high noise levels, effectively removing a significant amount of noise from the 3D point cloud surface, with only a few outliers remaining. These results demonstrate the strong robustness of the proposed method. 

### 5.4. Comparison with Competing Methods

This section focuses on analyzing the experimental results by comparing them with other methods, both subjective and objective, using a dataset of cultural relics obtained using a 3D scanner and three public 3D point clouds. To validate the superiority of the proposed method, we compared it with MRPCA [16], LR [11], the method proposed in [31], the method proposed in [19], and the method proposed in [24] in our experiments.

#### 5.4.1. Subjective Assessment

**Figure 12:**
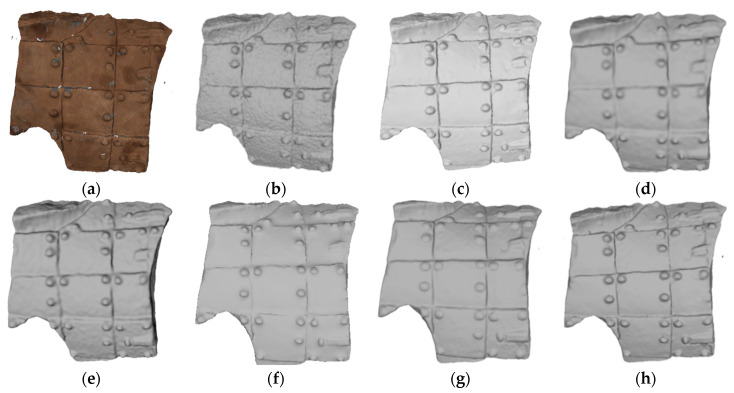
Denoising effect of several methods on G10-11-43(47)4: (**a**) description of ground truth; (**b**) description of noisy input; (**c**) description of our method; (**d**) description of MRPCA method; (**e**) description of the method in [31]; (**f**) description of LR method; (**g**) description of the method in [19]; (**h**) description of the method in [24].

Figure 12 shows the denoising results using different methods on the 3D fragment numbered G10-11-43(47)4. It can be seen from Figure 12f,g that both LR [11] and the method described in [19] effectively remove surface noise from the armor of the terracotta warriors. However, these methods also result in the smoothing of sharp features such as the rivets on the armor. Figure 12d,e show that the methods mentioned in [16,31] manage to better preserve the fine features of the rivets, but the surface of the armor still remains rough and uneven, and the noise is not completely removed.

The method proposed in [24] successfully eliminates surface noise while preserving the surface decoration of cultural relics, as shown in Figure 12h. In particular, our denoising method ensures the clear visibility of the rivets on the armor and achieves a satisfactory smoothing effect on the model surface, as shown in Figure 12c. The resulting 3D model, after noise removal, closely resembles the real cultural relic.

**Figure 13:**
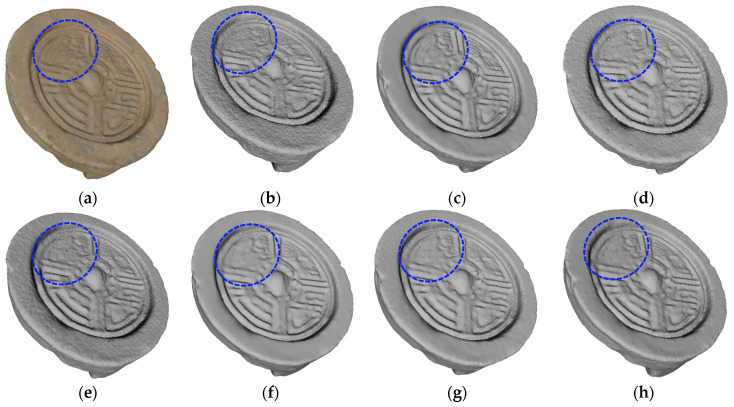
Denoising effect of several methods on Q002789: (**a**) description of the ground truth; (**b**) description of the noisy input; (**c**) description of our method; (**d**) description of MRPCA method; (**e**) description of the method in [31]; (**f**) description of LR method; (**g**) description of the method in [19]; (**h**) description of the method in [24].

The denoising effects of different methods on Q002789 are shown in Figure 13. Although the methods in Figure 13d,e can remove most of the noise on the surface of the 3D point cloud, there is still a small amount of noise attached to the surface that has not been removed. The denoising effect of the methods in Figure 13f–h is better than that of the methods in Figure 13d,e, but the pattern in the blue dotted circle is very blurred. In Figure 13c, the denoising effect of the proposed method is the most ideal and most similar to the real cultural relics, especially the area enclosed by the blue dotted circle, whose fine details are completely preserved.

**Figure 14:**
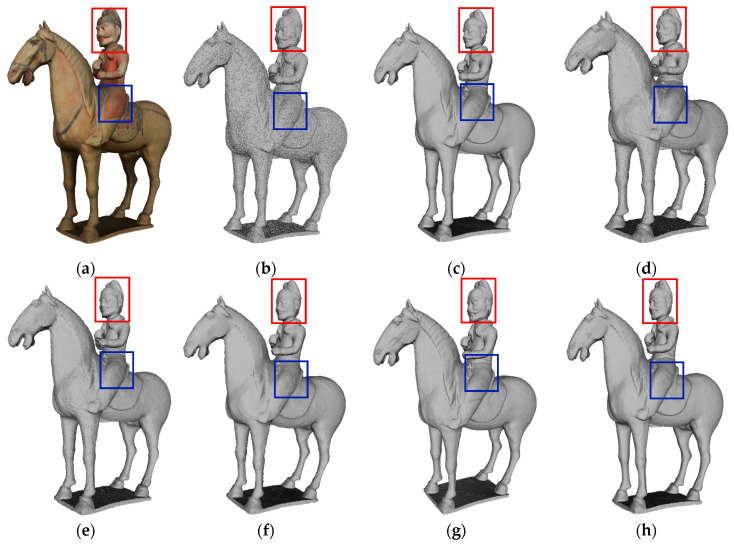
Denoising effect of several methods on H73: (**a**) description of the ground truth; (**b**) description of the noisy input; (**c**) description of our method; (**d**) description of MRPCA method; (**e**) description of the method in [31]; (**f**) description of LR method; (**g**) description of the method in [19]; (**h**) description of the method in [24].

The denoising effect of different methods on H73 is shown in Figure 14. It can be seen that, when using the denoising methods in Figure 14d,e, the surface of the sculpture of Hu terracotta army is uneven; in particular, the denoising of the face is insufficient, resulting in blurred facial contours and features. Evidently, the smoothing effect of the method in Figure 14f is better than that in Figure 14d,e. The methods described in Figure 14c,g,h overall show better performance compared to the methods in Figure 14d–f. These methods effectively enhance the clarity of facial contours and features in the Hu terracotta sculpture. For example, the details of the eyes and beards are well preserved. The method in Figure 14g is the worst of the three methods because there is still a small amount of noise on the surface that has not been completely removed. Both our method shown in Figure 14c and the method shown in Figure 14h demonstrate advanced denoising effects. It is important to highlight that the area encircled by the blue box is the hem of the dress, and several other competing methods produce unsatisfactory denoising results for this specific area, whereas our method successfully preserves the fine details of this dress.

**Figure 15:**
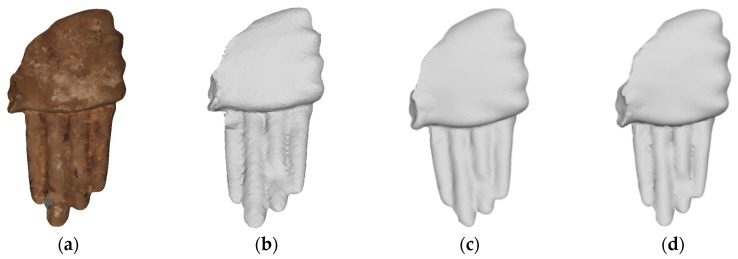

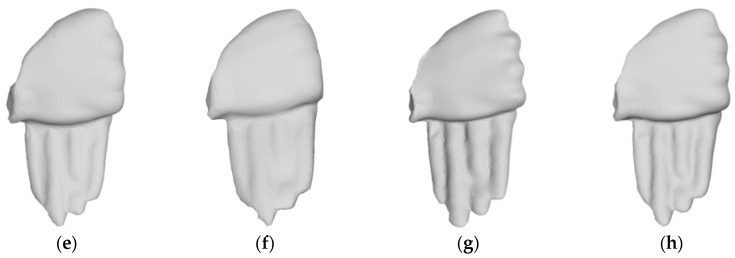
Denoising effect of several methods on G3-I-C-94: (**a**) description of ground truth; (**b**) description of noisy input; (**c**) description of our method; (**d**) description of MRPCA method; (**e**) description of the method in [31]; (**f**) description of LR method; (**g**) description of the method in [19]; (**h**) description of the method in [24].

The denoising results of different methods on the 3D fragment numbered G3-I-C-94 are shown in Figure 15. From Figure 15e,f, it can be seen that the finger part is too smooth after applying the denoising techniques proposed in [11,31]. Figure 15h shows that the method in [24] successfully preserves the intricate features of the palm and finger joint after noise removal. In Figure 15d,g, MRPCA [16] and the method in [19] effectively remove most of the noise, albeit with a slightly coarse denoising effect. Figure 15c shows that our proposed method is able to remove the noise substantially while still preserving the fine features of the finger cracks.

#### 5.4.2. Objective Assessment

We evaluated the proposed approach on cultural relic point clouds. To evaluate the denoising results, we introduced noise of different intensities into the clean 3D point cloud and quantitatively analyzed the results using the MSE and SNR. The experimental results in Table 4 and Table 5 show that as the noise intensity increases, the mean square error between the denoised point cloud and the clean point cloud also increases. When considering the EMS or SNR, our proposed method outperforms other competing denoising methods in terms of denoising effectiveness. Furthermore, even in the presence of high-level noise, our method maintains a small deviation between the two metrics, indicating its strong robustness. 

## 6. Conclusions

The acquisition of cultural relic point clouds can be achieved directly using 3D scanning equipment. However, this process is often imperfect, resulting in noise corruption in the point clouds. Removing noise from the surface of the cultural relic point cloud while preserving sharp details is a challenging task. To address this problem, we proposed an approach that combines color and geometric features to denoise the cultural relic point cloud. Our approach is based on graph signal processing, in which we formulated the denoising process as a minimization of graph Laplacian regularization. Utilizing color and geometric characteristics as signals, we approached the elimination of surface noise as an optimization dilemma with a graph signal smoothness prior. To evaluate the effectiveness of our denoising approach, we applied it to 3D cultural relic point clouds. It is important to highlight that the proposed approach is versatile and can be used in different applications where data are limited. The experimental results show that our approach outperforms five competing methods, effectively removing noise from the surface of cultural relic point clouds while preserving important details such as texture and ornamentation to a great extent.

## Figures and Tables

**Table 1:** Parameter setting.

Parameter	Down Sampling Rate	The Number of Points in the Patches	The Number of Nearest Neighbors of a Patch	$C^{*}$
Value	0.3	9	10	3

**Table 2:** Comparison of the proposed approach using only geometry and combined geometry and color on 4#yt.

Method	*σ*	Points	SNR (DB)	MSE	Iterations	Running Time (s)
Only geometry	0.02	58,380	60.66	0.61	4	172
Geometry + color			63.98	0.50	4	171
Only geometry	0.05	58,380	61.91	0.84	10	864
Geometry + color			62.14	0.72	11	870
Only geometry	0.1	58,380	59.50	1.07	46	3985
Geometry + color			62.03	0.81	49	4011
Only geometry	0.2	58,380	52.98	2.05	100	8884
Geometry + color			54.02	1.90	106	9003

**Table 3:** Comparison of experimental results before and after point cloud simplification on 4#yt.

Method	*σ*	Points	SNR(DB)	MSE	Iterations	Running Time (s)
Geometry + color	0.02	58,380	63.98	0.50	4	171
Geometry + color + simplification		30,000	64.12	0.55	1	42
Geometry + color	0.05	58,380	62.14	0.72	11	870
Geometry + color + simplification		30,000	61.98	0.76	2	139
Geometry + color	0.1	58,380	62.03	0.81	49	4011
Geometry + color + simplification		30,000	59.96	0.96	8	293
Geometry + color	0.2	58,380	54.02	1.90	106	9003
Geometry + color + simplification		30,000	51.99	1.89	29	1104

**Table 4:** MSE metric comparison of six methods using cultural relic data.

Data	*σ*	MRPCA	LR	[19]	[31]	[24]	Our Method
G10-52	0.02	0.697	0.760	0.684	0.704	0.650	0.630
G10-46-5	0.02	0.885	0.955	0.879	0.906	0.827	0.801
G3-I-C-94	0.02	0.797	0.842	0.774	0.801	0.746	0.769
G10-11-43(47)	0.02	0.658	0.678	0.633	0.659	0.613	0.573
Q002789	0.02	0.251	0.247	0.297	0.231	0.223	0.198
Q003418	0.02	0.503	0.515	0.472	0.497	0.421	0.432
H73	0.02	0.390	0.387	0.350	0.356	0.335	0.305
H80	0.02	0.567	0.581	0.549	0.545	0.534	0.521
Average		0.594	0.621	0.580	0.587	0.544	0.529
G10-52	0.03	0.801	0.826	0.799	0.819	0.759	0.733
G10-46-5	0.03	1.000	1.031	0.964	1.001	0.939	0.909
G3-I-C-94	0.03	0.940	0.948	0.899	0.921	0.873	0.826
G10-11-43(47)	0.03	0.719	0.730	0.687	0.713	0.659	0.642
Q002789	0.03	0.304	0.315	0.279	0.281	0.274	0.270
Q003418	0.03	0.579	0.576	0.561	0.570	0.554	0.542
H73	0.03	0.375	0.386	0.368	0.360	0.354	0.346
H80	0.03	0.634	0.641	0.639	0.619	0.624	0.620
Average		0.658	0.670	0.638	0.648	0.619	0.601
G10-52	0.04	0.948	0.945	0.915	0.930	0.872	0.820
G10-46-5	0.04	1.034	1.079	1.027	1.014	1.042	0.961
G3-I-C-94	0.04	1.032	1.045	0.984	1.023	0.987	0.992
G10-11-43(47)	0.04	0.741	0.734	0.717	0.722	0.699	0.641
Q002789	0.04	0.324	0.310	0.298	0.291	0.283	0.279
Q003418	0.04	0.649	0.655	0.634	0.627	0.618	0.609
H73	0.04	0.412	0.420	0.421	0.428	0.431	0.447
H80	0.04	0.749	0.740	0.759	0.733	0.711	0.723
Average		0.736	0.741	0.719	0.721	0.705	0.684
G10-52	0.05	0.943	1.157	0.911	0.921	0.907	0.857
G10-46-5	0.05	1.167	1.370	1.143	1.110	1.177	1.009
G3-I-C-94	0.05	1.097	1.265	1.024	1.057	1.103	0.983
G10-11-43(47)	0.05	0.867	0.948	0.833	0.802	0.756	0.709
Q002789	0.05	0.331	0.333	0.321	0.325	0.312	0.301
Q003418	0.05	0.729	0.733	0.702	0.711	0.697	0.687
H73	0.05	0.533	0.557	0.519	0.524	0.505	0.495
H80	0.05	0.802	0.823	0.785	0.799	0.791	0.779
Average		0.809	0.898	0.780	0.781	0.781	0.728
G10-52	0.1	1.158	1.188	1.199	1.149	1.045	0.941
G10-46-5	0.1	1.786	1.797	1.667	1.736	1.594	1.494
G3-I-C-94	0.1	1.433	1.453	1.338	1.421	1.281	1.105
G10-11-43(47)	0.1	1.185	1.195	1.164	1.177	1.152	0.912
Q002789	0.1	0.425	0.439	0.415	0.411	0.409	0.401
Q003418	0.1	0.749	0.750	0.736	0.741	0.730	0.724
H73	0.1	0.551	0.536	0.534	0.540	0.528	0.526
H80	0.1	1.103	1.101	1.098	1.067	1.076	0.997
Average		1.049	1.057	1.019	1.030	0.977	0.888

**Table 5:** SNR metric comparison of six methods using cultural relic data.

Data	*σ*	MRPCA	LR	[19]	[31]	[24]	Our Method
G10-52	0.02	60.10	59.53	59.86	60.99	61.29	62.33
G10-46-5	0.02	66.84	65.79	66.12	64.82	67.73	67.95
G3-I-C-94	0.02	61.03	58.55	61.85	61.42	62.28	62.24
G10-11-43(47)	0.02	66.02	65.88	66.57	65.81	67.21	67.23
Q002789	0.02	57.49	57.43	58.02	57.87	58.26	61.23
Q003418	0.02	62.68	62.70	63.74	66.34	64.54	66.21
H73	0.02	67.86	68.15	70.23	69.31	70.70	72.23
H80	0.02	71.01	69.43	71.26	71.85	73.22	73.22
Average		64.13	63.43	64.71	64.80	65.65	66.58
G10-52	0.03	58.90	58.47	59.03	58.48	59.68	59.78
G10-46-5	0.03	65.57	64.59	65.55	64.95	66.34	66.45
G3-I-C-94	0.03	59.85	59.41	59.88	59.52	61.65	61.49
G10-11-43(47)	0.03	65.54	65.46	66.02	65.83	66.51	66.99
Q002789	0.03	54.23	53.12	56.89	56.12	57.59	59.31
Q003418	0.03	60.43	60.95	61.98	62.35	62.13	62.87
H73	0.03	67.85	68.15	69.85	69.87	70.12	73.01
H80	0.03	69.27	68.99	70.56	70.04	71.23	72.01
Average		62.71	62.39	63.72	63.40	64.41	65.24
G10-52	0.04	57.79	57.37	57.64	57.93	58.25	58.41
G10-46-5	0.04	64.87	64.19	64.60	64.77	65.40	65.51
G3-I-C-94	0.04	58.80	58.77	58.95	59.01	59.37	59.32
G10-11-43(47)	0.04	65.60	64.92	65.22	65.08	66.03	66.13
Q002789	0.04	55.46	56.12	56.13	56.98	57.26	58.72
Q003418	0.04	58.57	58.66	59.02	59.54	60.03	61.23
H73	0.04	66.72	66.39	67.98	67.55	68.31	69.84
H80	0.04	66.54	66.23	66.75	67.12	67.00	67.88
Average		61.79	61.58	62.04	62.25	62.71	63.38
G10-52	0.05	57.14	56.87	56.75	57.08	57.82	57.96
G10-46-5	0.05	63.78	63.34	63.46	63.57	63.70	63.77
G3-I-C-94	0.05	58.45	57.67	58.25	58.13	59.05	59.11
G10-11-43(47)	0.05	64.27	63.33	64.36	64.56	65.07	65.11
Q002789	0.05	54.64	55.03	56.01	55.46	56.87	56.99
Q003418	0.05	56.44	55.85	56.77	57.03	57.98	58.34
H73	0.05	66.75	66.23	67.41	67.06	66.58	67.56
H80	0.05	63.96	63.18	64.85	64.86	64.23	65.01
Average		60.68	60.19	60.98	60.97	61.41	61.73
G10-52	0.1	54.85	54.93	54.85	54.93	55.00	55.09
G10-46-5	0.1	59.16	59.33	59.15	59.22	59.22	59.30
G3-I-C-94	0.1	55.52	55.29	55.50	55.51	55.52	55.60
G10-11-43(47)	0.1	59.55	59.43	59.54	59.48	59.57	59.66
Q002789	0.1	52.45	52.32	54.51	53.56	53.77	54.90
Q003418	0.1	53.54	53.85	53.67	53.30	53.82	53.14
H73	0.1	63.57	63.33	63.47	63.06	63.83	62.62
H80	0.1	60.86	60.23	61.77	61.66	61.29	62.34
Average		57.44	57.34	57.81	57.59	57.75	57.83

## Data Availability

Data are contained within the article.
